# A Spectral-Domain-OCT-Guided One-Year Follow-Up of Newly Diagnosed Pediatric Idiopathic Intracranial Hypertension Patients

**DOI:** 10.3390/diagnostics16030457

**Published:** 2026-02-01

**Authors:** Yuval Cohen, Michael Eidel, Aviv Vidan, Gilad Hadar, Otzem Chassid

**Affiliations:** 1 Department of Ophthalmology, Ziv Medical Center, Safed 1311001, Israel; aviv.vidan@gmail.com (A.V.); gilad6709@me.com (G.H.); otzem73@gmail.com (O.C.); 2 Azrieli Faculty of Medicine, Bar-Ilan University, Safed 1311502, Israel; 3 Department of Ophthalmology, Hillel Yaffe Medical Center, Hadera 3820302, Israel; 4 Pediatric Neurology and Child Development Institute, Hillel Yaffe Medical Center, Hadera 3820302, Israel; eidel.mich@gmail.com

**Keywords:** pseudotumor cerebri, idiopathic intracranial hypertension, retinal nerve fiber layer, optical coherence tomography

## Abstract

**Background/Objectives:** To examine longitudinal changes in total retinal nerve fiber layer thickness (RNFLT) as the primary outcome measure in newly diagnosed pediatric idiopathic intracranial hypertension (IIH) patients using Spectral-Domain Optical Coherence Tomography (SD-OCT) at one-year follow-up. **Methods:** This is a prospective observational cohort study with cross-sectional control-group comparison. We included children with clinically definite IIH (IIH group) and children without papilledema and a normal neurological exam as a control group. Optic nerve parameters, including the primary outcome measure RNFLT and secondary outcome measures such as total retinal thickness (TRT) and optic disk area (ODA), were evaluated using SD-OCT (3D OCT-2000, Topcon, Topcon Corporation, Tokyo, Japan). Evaluations took place at presentation and, for the IIH group, before lumbar puncture (LP), at 1-day post-LP and at 1-, 3-, 6-, and 12-month follow-ups. **Results:** A total of 44 children aged 7–17 years were recruited (IIH group: N = 19, control group: N = 25). The mean baseline RNFLT was 133.1 ± 18.5 µm and 113.1 ± 8.7 µm for the IIH and control groups (*p* < 0.001), respectively. The IIH group showed a significant decline in RNFLT at the third-month follow-up. Between 3-month to one-year follow-up, mean total RNFLT showed an insignificant decline of 6 µm and did not differ from the RNFLT of the control group; however, segmental analysis of RNFLT showed a significant decline in the thickness of the nasal segments. At the one-year follow-up, two children had significant thinning of RNFLT at the superior quadrant. Intracranial pressure measured in the IIH group was directly correlated with RNFLT at the superior segment. **Conclusions:** SD-OCT is a useful non-invasive adjunct tool for the diagnosis and follow-up of IIH in children from primary school age onward. RNFL thickening resolved in most children at 3 months from IIH diagnosis. The study is constrained by specific methodological limitations, including a small sample size and non-contemporaneous evaluation of the control group compared with the IIH group. The significance of the segmental RNFL changes observed after one year should be further investigated with regard to long-term development, if possible with a larger prospective study that also considers the ganglion cell layer to explore for permanent axonal damage to the optic nerve.

## 1. Introduction

Pediatric idiopathic intracranial hypertension (IIH) is defined as raised intracranial pressure (ICP) in the absence of a known etiology [1]. IIH is a rare condition that can have deleterious effects on vision, and if undetected and untreated, can lead to permanent visual impairment [2,3]. Lumbar puncture (LP), an invasive diagnostic procedure measuring ICP in children at risk of intracranial hypertension, remains the gold standard for confirming IIH [4]. Non-invasive diagnostic tools such as fundoscopy, radiography, and ocular ultrasonography lack adequate sensitivity to be used as screening tools [5].

Pediatric IIH induces optic nerve head and retinal changes, which can be detected and quantified through ultrahigh-resolution cross-sectional images using Spectral-Domain Optical Coherence Tomography (SD-OCT) [6,7]. OCT imaging of the optic nerve and retina has demonstrated significant diagnostic value in pediatric IIH cases. In a retrospective study, OCT was shown to assist in differentiating mild papilledema from pseudopapilledema when measuring the transverse horizontal diameter of the opening in Bruch’s membrane [8]. Other studies showed that children with IIH had a thicker-than-average RNFL as compared to normal controls, and suggested that OCT can be used as a supplementary method to aid in the reliable detection of papilledema [9,10]. Furthermore, RNFL thinning detected by SD-OCT was strongly associated with peripheral vision loss, central or paracentral scotoma, and general vision loss [11]. A review study of pediatric IIH suggested that OCT could be used not only to aid in diagnosis of children unable to perform field tests, but also as a surrogate monitoring marker correlating with ICP [5]. Another systematic review proposed an opposing view to the routine use of OCT in IIH and concluded that the widespread use of OCT to detect an elevated ICP in IIH children as standard clinical practice was not recommended due to non-verified diagnostic accuracy [12]. Moreover, several studies showed inconsistencies in OCT measures of the optic nerve and retina partially due to a mixed study population of both newly diagnosed and already known IIH [13,14].

There are several downsides when using OCT in a young child. First, age-specific adjustments in the OCT imaging protocol need to be established in order to ensure optimal image acquisition [15]. Second, normative values for RNFL thickness are not available for children. Moreover, there is an inherent obstacle in using conventional SD-OCT to evaluate pediatric IIH, since a chin rest system requires good fixation and cooperation, thus limiting its use in very young children [8,9,16,17]. Treatment strategies in the pediatric population mostly follow approaches used in adults, since no randomized controlled trials in children are available to date [18].

In adults, OCT was shown to be a valuable objective tool to monitor patients during a 3-month period after diagnosis of IIH [19]. A randomized control study in adults with acute IIH showed that RNFL and TRT thicknesses as well as ONH volume measurements proved useful when evaluating treatment of papilledema during a 6-month follow-up [20].

At preschool age, child cooperation improves and ocular biometry reaches adult values. In a 5-year-old child, ocular biometric properties result in 5% OCT image magnification, and after the age of 10 years, there should be no OCT image adjustments [15]. Utilizing these benefits, the goal of our study was to examine the changes in optic nerve parameters in newly diagnosed idiopathic intracranial hypertension (IIH) in children of primary school age and beyond, using SD-OCT during a one-year follow-up.

## 2. Methods

This prospective observational study compared the optic nerve SD-OCT (3D OCT-2000, Topcon, Topcon Corporation, Japan) images of 19 children newly diagnosed with IIH to 25 controls. The study was approved by Hillel Yaffe Medical Center Ethics Committee (protocol code HYMC-0027-16; date 15 May 2016) and was performed in accordance with the guidelines of the Declaration of Helsinki of the World Medical Association. The trial was registered at the NIH (ClinicalTrials.gov identifier: NCT02665858; registration date: 24 January 2016). Written informed consent was obtained from all participants’ legal guardians.

**Figure 1:**
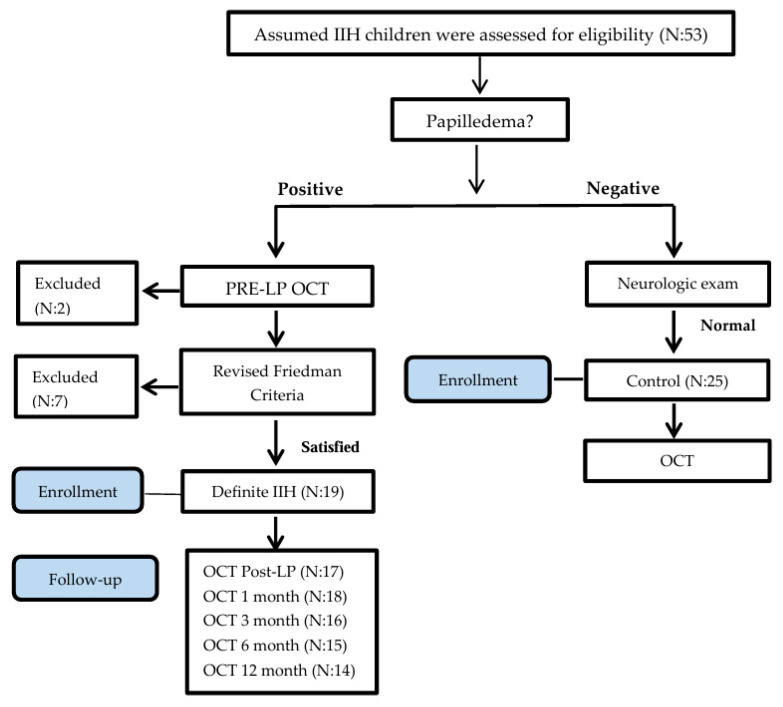
Consort flow chart.

As a common practice, many children are referred for ophthalmologic and neurologic exams to Hillel Yaffe Medical Center by outpatient neurologists, ophthalmologists, and pediatricians to rule out suspected papilledema in cases of assumed IIH. Children ages 7 to 17 years were assessed for eligibility (Figure 1) in the study from May 2016 to April 2018 (23-month enrollment period). Initially, children underwent a complete ophthalmologic assessment including an exam for the presence of disc swelling by two senior ophthalmologists. A complete physical exam was performed by a pediatrician, and a neurologic exam by a certified pediatric neurologist. The presence of a unilateral or bilateral Abducens nerve palsy and chronic headache were recorded.

In the presence of disc swelling confirmed by two ophthalmologists, inclusion into the IIH group for the study required fulfillment of the Friedman criteria [4]. The degree of papilledema was determined by frequency distributions of Frisén grade based on slit lamp examination. Children positive for disc swelling underwent a CT/MRI brain scan to rule out a brain lesion or other neurologic etiology for disc swelling. Normal imaging was then followed by lumbar puncture (LP) for measurement of ICP and cerebrospinal fluid evaluation.

### 2.1. Intracranial Pressure Measurements

The child was placed in a lateral decubitus position with legs extended, head and spine horizontal, and as relaxed as possible. We used a standard 18-gauge spinal needle and a manometer positioned at a 90-degree angle to the patient’s spine. The fluid column in the manometer was allowed to rise until it stopped and fluctuated slightly with the patient’s breathing. The ICP was read at the lowest point of the fluid’s meniscus. Subsequently, 10 mL CSF was removed for further analysis. Elevated ICP was defined as >25 cmH_2_O in a non-obese child and ICP > 28 cmH_2_O in an obese child.

Control children were selected from the same referral population and defined as having the following:

1. An absence of papilledema on dilated fundoscopic examination.
2. A normal physical examination by pediatrician.
3. A normal neurological examination by certified pediatric neurologist.
4. No history of chronic headaches with associated neurological signs.
5. No documented abnormalities on prior neuroimaging, if performed clinically.

Exclusion criteria included the following:

1. Ophthalmologic diseases that could influence OCT imaging, such as corneal disease, glaucoma, uveitis, optic nerve hypoplasia, or retinal disease.
2. Children with secondary IIH (as per Revised Friedman Diagnostic Criteria) [4].
3. Developmental delay or behavioral disorder precluding reliable cooperation with OCT image acquisition.

For both the IIH and control groups, the following data were compiled: disease duration, presenting symptoms (i.e., headache, transient visual obscurations, tinnitus, dizziness, nausea, vomiting, diplopia, photophobia), current medical treatment, and obesity evaluation (height, weight, BMI). The obesity evaluation was based on the gender-specific BMI-for-age growth charts [21]. Presence of puberty was evaluated by family anamnesis and/or observed presence of secondary sexual characteristics.

### 2.2. Medical Treatment for IIH Group

Acetazolamide (carbonic anhydrase inhibitor) was initiated in all patients (15 mg/kg/day) and divided into twice-daily dosing (typical starting dose: 250 mg twice daily). Over the course of two weeks, this dosage was increased to a maximum level ranging from 500 mg to 2000 mg, with specific adjustments made based on the patient’s clinical symptoms, side effects, and tolerance of the medication. In instances of severe papilledema or an inadequate response to acetazolamide alone, combination therapy was considered with furosemide (40 mg daily). The total treatment plan consisted of a three-month active phase followed by a three-month tapering period, overseen by a neurologist. Additionally, weight reduction was established as a primary therapeutic goal, with all obese patients receiving counseling regarding weight loss.

Documented Treatment Parameters

1. Initial acetazolamide dose in mg/kg
2. Maximum dose achieved
3. Duration of treatment
4. The use of any adjunctive furosemide therapy
5. Patient adherence to the regimen, as reported by a guardian

### 2.3. Visual Field

The Humphrey^®^ Field Analyzer (HFA™, Carl Zeiss Meditec AG, Jena, Germany) with central 24-2 protocol and the SITA-Standard protocol were used for the visual field (VF) exam. The VF was performed only in cooperative children in the IIH group during follow-up.

### 2.4. SD-OCT Protocol

Scans of the right and left eyes were obtained using an SD-OCT machine (3D OCT-2000, Topcon) at pre-LP, 1-day post-LP, and at 1-, 3-, 6-, and 12-month follow-ups. All scans were acquired by trained technicians. Two individual optic disc cube 6 × 6 mm^2^ protocol scans were obtained for each subject after pupil dilatation, and the best-image-quality scan was selected for analysis. This protocol is based on a three-dimensional scan centered on the optic disc, where information from 512 (depth) × 200 × 200 volume scans with an axial resolution of 5 µm is collected. The optic disc center was automatically detected, and a 3.4 mm diameter circle, which consisted of 1024 A-scans, was placed around the optic disc center. To be included, all images were reviewed and had to have an image quality score > 40 and the absence of movement artifacts [22]. The built-in analysis software (version 7.11) automatically segmented the RNFL, total retinal thickness (TRT), and optic disc area (ODA) boundaries and calculated their thickness. Conventional circumpapillary RNFL/TRT measurements on the 3.4 mm diameter circle were taken and averages were computed for 4 and 12 segments: quadrants Q_1_ (superior quadrant; segments_11,12,1_), Q_2_ (inferior quadrant; segments_5,6,7_), Q_3_ (nasal quadrant; segments_2,3,4_), Q_4_ (temporal quadrant; segments_8,9,10_), and hourly segments S_1_–S_12_. RNFLT, TRT, and ODA were evaluated for longitudinal changes.

### 2.5. Primary and Secondary Outcome Measures

The primary outcome measure was RNFLT at baseline and serial follow-up intervals (1, 3, 6, 12 months).

The secondary outcome measures were TRT and ODA.

### 2.6. Data Analysis

We determined the minimum sample size needed to differentiate between the IIH and control groups as 22 (11 in each group) based on pilot data with anticipated value of outcome in the IIH group for RNFL thickness of 130–140 µm, RNFL thickness of the control groups of 110 µm, standard deviation of outcome in population of 15 µm, significance of 0.05, power level of 0.80, and dropout rate of 10%. The descriptive analysis used mean, standard deviation, and range for continuous variables. Counts and percentages were reported for categorical variables. Differences between the groups in demographic and SD-OCT parameters were analyzed by using a *t*-test. An independent-samples *t*-test was used to assess between-group differences (IIH vs. control) at baseline (total, quadrant, and segmental RNFLT, TRT, and ODA). A repeated-measures ANOVA followed by pairwise analysis was used to assess longitudinal within-group changes in the IIH group over time. To calculate the adjusted *p*-value (*p*_adj_), the Bonferroni correction was applied to the original (raw) *p*-values derived from each pairwise analysis. This adjustment was performed by multiplying the raw *p*-value by six, representing the total count of distinct time points tested: prior to the lumbar puncture (pre-LP), one day following the procedure, and at follow-up intervals of 1, 3, 6, and 12 months. Associations between continuous variables were described using Pearson correlation coefficients. Statistical significance was set at *p* < 0.05 and statistical analysis was carried out by using SPSS (version 26.0; IBM Corp., Armonk, NY, USA).

## 3. Results

Fifty-three children were assessed for eligibility in the study. Excluded children (Figure 1) were due to noncompliance with the LP examination (N = 1), retinal disease (retinitis pigmentosa; N = 1), and failure to fulfill the Revised Diagnostic Criteria for pseudotumor cerebri syndrome in adults and children (N = 9).

Forty-four children were included, and their demographics are displayed in Table 1.

The mean age of the IIH group was 12.9 ± 2.9 years, which was comparable to the control group. Nearly half (47.4%) of the children in the study group were of prepubertal age. Children newly diagnosed with IIH had an average ICP of 37.3 ± 12.5 cmH_2_O; notably, 42% had severely elevated ICP, defined as being greater than 40 cmH_2_O.

The clinical presentation of patients upon first examination is presented in Table 1. A significant difference in visual acuity (VA) was measured between the IIH and the control groups. The IIH group had two children with a moderate decline in VA (the first with 20/40 had a pre-LP RNFLT of 136 µm and second child had a VA of 20/80 with a pre-LP RNFLT of 155 µm). In the control group, visual acuity was measured at 20/20, except for three children with a VA of 20/25. Headache was the most frequent complaint presented by patients upon their first examination, occurring in both the IIH group and the control group. Prepubertal children diagnosed with IIH had a comparable number of symptoms as compared to the control group (2.6 + 1.9 vs. 3.5 + 2.0, respectively; *p* = 0.35). When headache was present in prepubertal children with IIH, it was typically accompanied by transient visual obscuration (TVO) or pulsatile tinnitus. A consistent finding among all newly diagnosed IIH children was the presence of bilateral swollen discs. The mean Frisén score recorded was 2.9 ± 1.1. One month after diagnosis, six patients were compliant with the visual field exam, and the measurement of their mean deviation in the worst eye was −6.8 ± 9.3 dB (range −1.49 to −24 dB).

### 3.1. Treatment Characteristics of IIH Group

Patients began treatment with acetazolamide at a dose of 15 mg/kg/day, which was gradually increased over two weeks to reach a maximum average of 18.8 ± 2.4 mg/kg/day. The medication was then tapered down starting at the three-month mark, resulting in a complete cessation of treatment by six months. One patient required a combination of furosemide and acetazolamide. Eighteen families reported good adherence to prescribed regimen.

### 3.2. SD-OCT Optic Nerve Parameters

OCT scans of the right eyes, presented in the figures, were taken at pre-LP (N = 19), 1–3 days post-LP (N = 17), and at 1- (N = 18), 3- (N = 16), 6- (N = 15), and 12-month (N = 14) follow-ups. OCT was scheduled at 24 h after LP procedure. Five children had a long recuperation time from the LP procedure, which delayed their OCT imaging by 2–3 days.

### 3.3. Primary Outcome: RNFL Thickness

**Figure 2:**
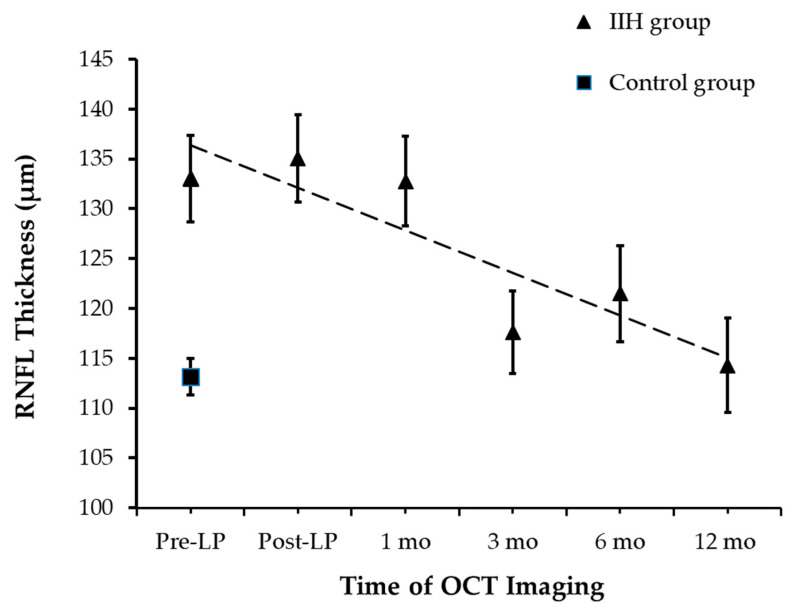
RNFL Thickness Changes During Follow-up.

At the time of presentation, RNFLT was measured successfully in all 44 children. RNFLT was 133.1 ± 18.5 µm [95% CI: 123.7 µm, 142.2 µm] and 113.1 ± 8.7 µm [95% CI: 109.2 µm, 130.1 µm] in the IIH and control groups, respectively (an independent-samples *t*-test, *p* < 0.0001; Figure 2). The removal of 10 mL of CSF did not affect RNFLT as post-LP RNFLT measurements were not significantly different from pre-LP RNFLT. A statistically significant decline in average RNFLT was observed over 12 months (repeated measures ANOVA, *p* < 0.017), though no significant differences in RNFLT were noted within the first month of follow-up (pairwise analysis for pre-LP vs. post-LP: *p*_adj_ = 1, and post-LP vs. 1 month: *p*_adj_ = 0.82).

A significant reduction in RNFLT was observed by the 3-month mark. At the 3-month follow-up, there was a significant decline in RNFLT of 18 µm when compared to the RNFLT measured at 1 month (*p*_adj_ = 0.03). Total RNFLT of the IIH group measured at 3 months was comparable to the control group at baseline (an independent-samples *t*-test, *p* = 0.25). Following the 3-month follow-up, the mean total RNFLT remained stable until 12-month follow-up.

The measurements tracked the RNFLT across a timeline from pre-procedure up to one year.

**Figure 3:**
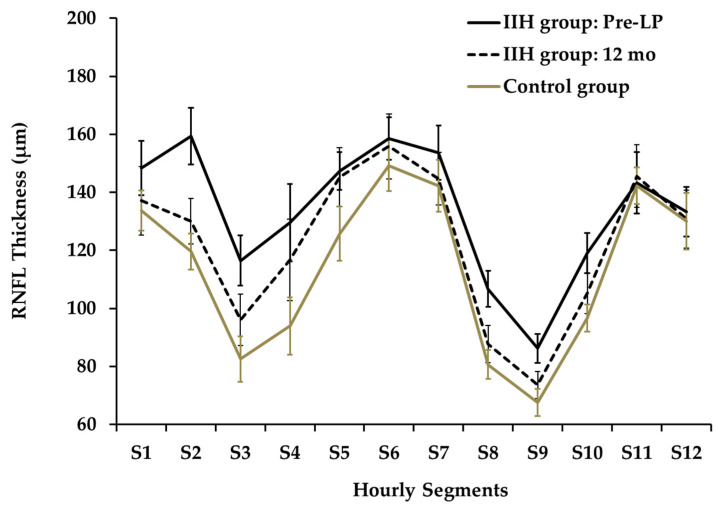
Hourly Segments of RNFL Thickness at Presentation and at 12 months.

The twelve-segment analysis of RNFLT (Figure 3) showed significant differences between mean pre-LP IIH and control groups at segments S_2,3,4,8,9,10_ (*p* < 0.001, 0.001, 0.01, 0.01, 0.001, 0.01, respectively).

**Figure 6:**
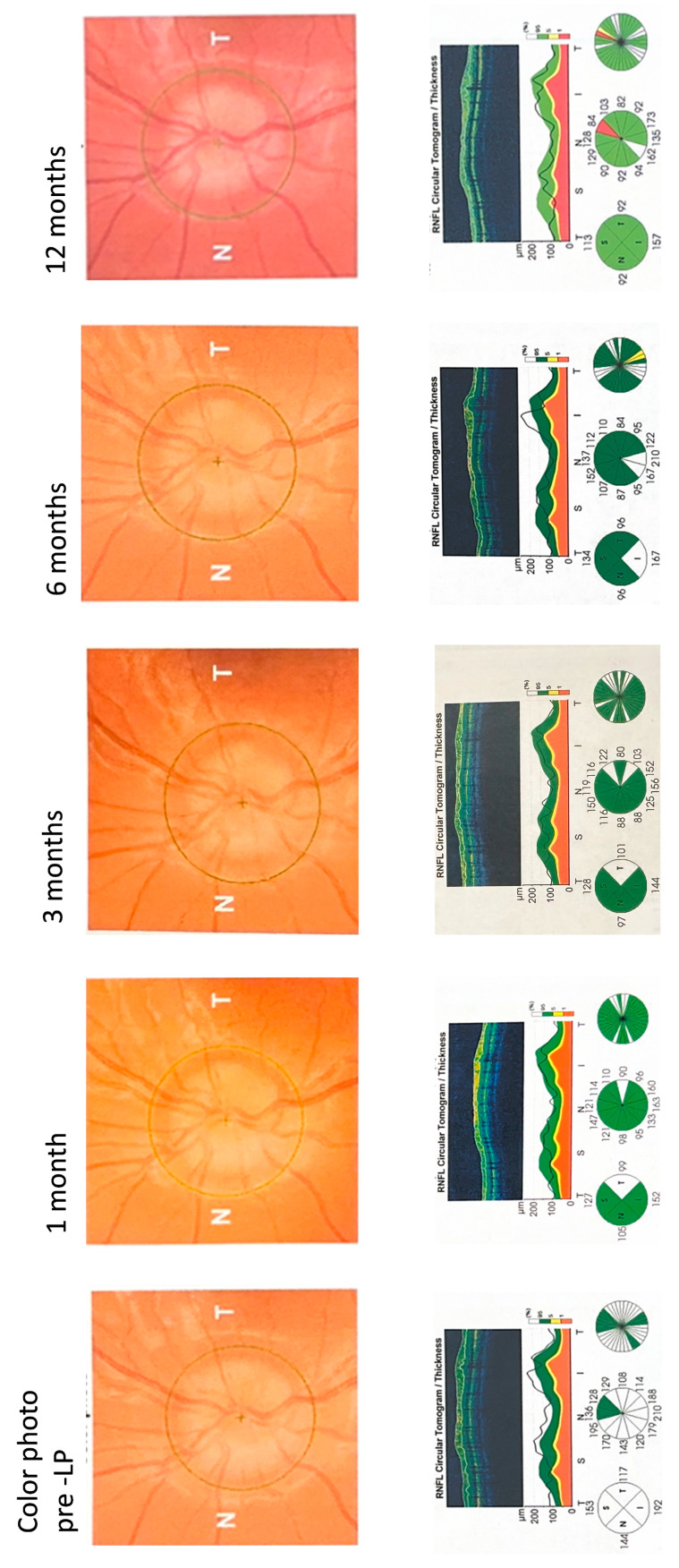
SD-OCT Optic Nerve Scans During Follow-up.

A segmental analysis performed at the 6-month follow-up demonstrated a significant reduction in thickness for segments S_2_ and S_3_ relative to the baseline measurements. Specifically, RNFLT for S_2_ decreased from 157.2 µm to 119.9 µm (*p*_adj_ = 0.005), while the thickness for S_3_ dropped from 114.2 µm to 90.4 µm (*p*_adj_ = 0.027). At the 12-month follow-up, the data highlighted outliers in specific subjects: two children presented with an RNFLT in Q_superior_ that was below the normal 5th percentile (e.g., presented in Figure 6), whereas three children displayed a mean RNFLT exceeding the 95th percentile.

At presentation, the nasal quadrant exhibited the greatest increase in RNFLT when compared to the control group, measuring 131.1 µm vs. 97.1 µm respectively (an independent-samples *t*-test, *p* < 0.0001).

### 3.4. Secondary Outcome Measures: TRT and ODA

**Figure 4:**
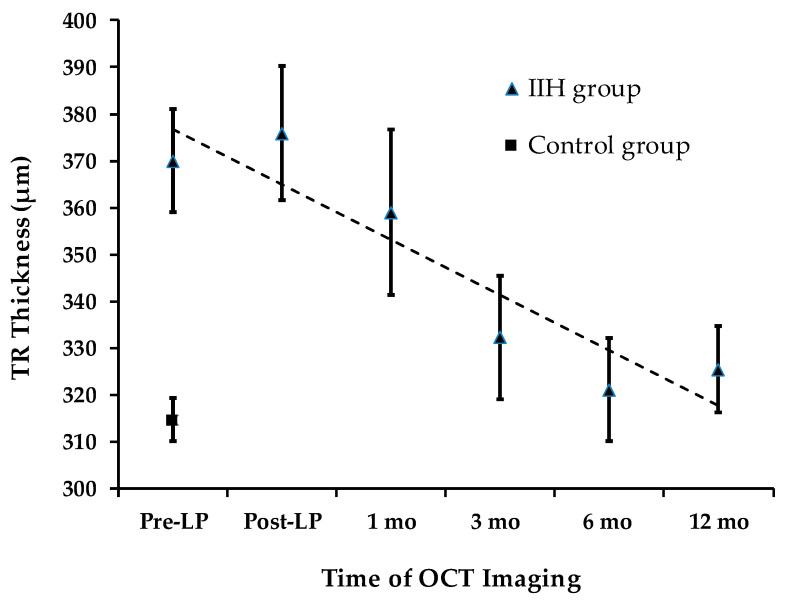
TRT Changes During Follow-up.

Total retinal thickness (TRT) longitudinal changes are presented in Figure 4. At presentation, TRT was significantly greater in the IIH group (373.6 ± 47.2 µm) compared to the control group (316.4 ± 21.7 µm) (an independent-samples *t*-test, *p* < 0.0001). Quadrant analysis of TRT also showed significant differences between the IIH and control groups.

### 3.5. TRT: Total Retinal Thickness

Over a 12-month period, a statistically significant decline in average TRT was observed (repeated measures ANOVA, *p* < 0.008). The mean total TRT measured pre-LP (373.6 ± 45.7 µm) was not significantly different from the level recorded at the 1-month follow-up (358.9 ± 70.9 µm) (*p*_adj_ = 0.93). This stability mirrors the trend observed in total RNFLT during the same timeframe. However, a significant decline in total TRT of 41.2 µm was detected at the 3-month follow-up (pairwise analysis for pre-LP vs. 3 months: *p*_adj_ = 0.027).

**Figure 5:**
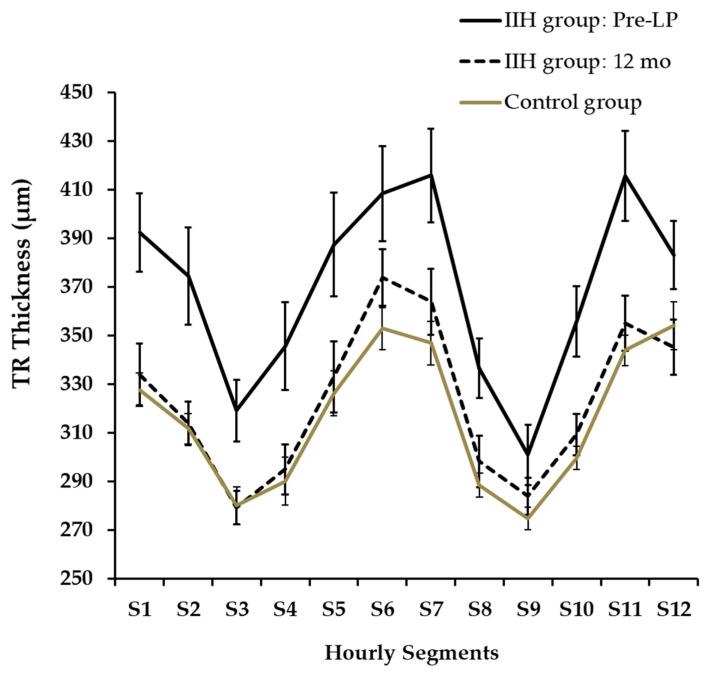
Hourly Segments of TRT at Presentation and at 12 Months.

Hourly segments of TRT at presentation and at 12 months are presented in Figure 5. TRT was not automatically segmented in three cases (N:2, S_12_; N:1, S_6_) and we did not further analyze these OCT images via manual segmentation. A twelve-segment analysis indicated that TRT was significantly thicker in the IIH group at pre-LP than in the control group across all segments (an independent-samples *t*-test, *p* < 0.02), with the exception of segments S_2_ and S_9._ Furthermore, when compared to the initial pre-LP levels, the IIH group exhibited a significant decrease in TRT after 12 months in all segments (*p*_adj_ < 0.04) except for S_6,12_.

ODA was not automatically segmented in two cases, and we did not further analyze these OCT images via manual segmentation. At presentation, ODA was 5.1 ± 1.9 mm^2^ for the IIH group and 2.77 ± 0.56 mm^2^ for the control group (an independent-samples *t*-test, *p* < 0.0001). Optic disc area (ODA) was the first parameter to show a reduction in size already at the one-month follow-up (4.0 ± 1.3 mm^2^; pairwise analysis for pre-LP vs. 1 month: *p*_adj_ < 0.0001). At 3 months, further decline in ODA was measured at 3.63 ± 0.86 mm^2^ (pairwise analysis for pre-LP vs. 3 months: *p*_adj_ = 0.007); however, ODA was larger than in the control group (an independent-samples *t*-test, *p* = 0.04). No significant changes were noted at the 6- and 12-month follow-ups.

### 3.6. Intracranial Pressure and RNFL/TRT/ODA Parameters

The segment S_2_ of pre-LP RNFL was directly correlated with ICP (r = 0.51, *p* = 0.03). The nasal and temporal quadrants of TRT had a moderate direct correlation with ICP (Q nasal r = 0.54 and Q temporal 0.49; *p* = 0.01 and 0.03, respectively). The TRT segments S_2,3,10_ had a direct correlation with ICP (r = 0.49–0.56, *p* < 0.05). ODA did not correlate with ICP (r = 0.37, *p* = 0.15). ICP measured at presentation was directly correlated with measured post-LP RNFL with S_2_, S_3_, and S_12_ and at 1 month with S_3_ and S_7_.

Figure 6 shows the left-eye SD-OCT optic nerve follow-up scans of a 14-year-old non-obese child with acute IIH presenting with a headache, transient visual obscuration, and tinnitus. A fundus exam showed that the major vessels were obscured by edema as they leave the disc, which corresponds to a Frisén score of 3. The patient’s brain imaging and neurologic exams presented as normal. The patient’s ICP was measured at 31 cmH_2_O and the cerebrospinal fluid constituents were normal. The child was treated with 500 mg of acetazolamide twice daily. The patient’s RNFLT measurements on the OCT scans fell within the white zone (above the normal green zone), indicating significant pre-LP swelling. The patient showed fast recovery in the RNFLT readings. At 12-month follow-up, RNFLT thinning was noted at the superior segment. 

## 4. Discussion

In the present study, we investigated the value of OCT for detecting papilledema and following optic nerve sequelae for one year in pediatric patients with IIH. At presentation, total RNFLT, TRT, and ODA were significantly greater than in controls. The optic nerve parameters measured in our control group were comparable to those of healthy control groups measured in previous pediatric IIH studies [9,10]. A significant small decline in visual acuity was measured in 31% of the children. We showed that RNFLT recovery occurs within a three-month period, specifically between the one-month and three-month follow-up appointments. At 12 months, two children had significant thinning in segmental RNFLT/TRT, indicating potential damage to the optic nerve.

To the best of our knowledge, this is the first preliminary study that used OCT measures to monitor recovery, with a one-year follow-up of acute pediatric IIH. A previous comparable study was performed in adult IIH, describing the changes in total RNFL of eight patients over 6 months [23]. Limiting factors for OCT imaging in children include patient motion, poor fixation, patient position, and different optics compared with the adult eye. Moreover, the interpretation of OCT in the setting of ongoing retinal and optic nerve development in young children is challenging [15]. In recent years, handheld OCT has simplified the examination of the optic nerve and retina for infants and young children and has been proven accurate in detecting an elevated ICP in idiopathic and secondary IH as presented in the RIO study (recognition of intracranial hypertension using handheld optical coherence tomography in children) [16]. In neonates and young children, OCT scans should be customized for the unique optical parameters of small eyes; however, in preschool-aged children, the expected optics are comparable to those of an adult [15]. We showed that serial high-quality images derived from conventional OCT performed on preschool children are plausible and assist in diagnosing and monitoring the progress of pediatric IIH. The establishment of age-specific normative developmental OCT data might enhance the accuracy of interpretation in pediatric OCT imaging.

Currently, there is limited knowledge about the natural history of pediatric IIH, and in most cases it has been reported to be a short episodic disease [5,24,25,26]. In our study, rapid resolution of RNFLT thickening occurred within 3 months when papilledema was moderate with a 2.9 mean Frisén score. In a recent retrospective cohort study, the mean time to clinical resolution was 2.2 months, while papilledema resolved after a mean of 7.8 months [27]. This rapid response distinguishes children from adults. In adults, the recovery of RNFL was longer, as 50% and 75% of the eight patients showed recovery of IIH after 3 and 6 months, respectively [23]. Disease recovery might be altered by various risk factors linked to disease severity such as adherence to medication, follow-up, and weight loss in obese children [27]. OCT enabled objective serial measurements of pediatric IIH that assisted in appreciating the evolution of changes in the optic nerve head during follow-up and supported the overall clinical work-up.

The application of OCT measures has mainly focused on the diagnosis of papilledema in children. Previous studies recommended obtaining a follow-up optic nerve evaluation and OCT scan to detect any significant change that supports the diagnosis of concomitant papilledema in a child with optic nerve drusen [1,28,29]. Moreover, the accuracy of OCT imaging to differentiate between papilledema and pseudopapilledema in children was challenged by two reviews [12,30]. The first study concluded that although SD-OCT demonstrated high positive-percent agreement and negative-percent agreement with a clinical diagnosis of papilledema in children, it cannot conclusively differentiate papilledema from pseudopapilledema in children because of the lack of high-quality evidence. On the contrary, another study performed in the United Kingdom showed that 95% of children who were examined by community optometric practices and were referred with suspected papilledema did not have papilledema. The authors suggested that OCT could be used as a screening tool, using filtering cut-offs measures to support papilledema and limiting the referrals to high clinical suspicion children [31]. Optimizing the decision threshold should help avoid diagnostic errors of papilledema that might carry a significant risk or lead to invasive, redundant, and costly testing.

Several studies reporting the correlation between ICP and OCT optic nerve parameters showed either no or a positive correlation [9,32,33]. The optic disk swelling in IIH is uneven, as optic nerve axonal fibers might be linked to hydrostatic, structural, and biological variables. Elevated hydrostatic pressure at the distal optic nerve sheath favors an even distribution of fluid; however, structurally dependent compression of axons as they exit the lamina cribrosa as well as stagnation-related toxicity of CSF might be responsible for uneven axonal swelling [6]. Moreover, in our study, ODA was not correlated with ICP, limiting its utility for non-invasive ICP monitoring. ICP was directly correlated with one segment of RNFLT and two quadrants of TRT. The possible explanations for our results include the following: First, papilledema of IIH is an evolving process. The delay in appearance of papilledema in acutely elevated ICP is consistent with the idea that papilledema is not based on dilated veins, but rather on the interruption of the metabolic process, which mediates axoplasmic flow. Second, RNFL thickening in papilledema is often not symmetric, with an increase in thickness favoring superior and inferotemporal sites, while temporal RNFL thickening is a finding occurring in association with more severe papilledema. Finally, in the pediatric age group, the reference range for cerebrospinal fluid (CSF) opening pressure varies greatly, with a normal range between 10 and 28 cmH_2_O. Clinical variables such as age, depth of sedation, and obesity may influence the ICP measurements and might significantly affect the correlation between ICP and RNFL [34].

There are several limitations to the present study. First, we included a small number of patients with definite IIH without treatment allocation concealment or blinding of outcome assessors. Therefore, causal inference regarding the effect of medical treatment on RNFL recovery cannot be established. The unequal and small group sizes (N = 19 IIH vs. N = 25 controls) and attrition in the IIH group follow-up further limit generalizability. Observed RNFL recovery may reflect natural disease course, treatment effects, or a combination of both; attribution is not possible from this design. Second, during recruitment, ICP was not measured and MRI was not performed in the control group; therefore, a diagnosis of IIH could theoretically still be suggested [4]. Third, we limit our conclusion to children of primary school age upwards, since handheld OCT was not available in our department to examine infants and younger children. Fourth, our study at a single-site tertiary referral center inherently includes a multidisciplinary approach that might influence the patient recovery pattern that we observed in our cohort. As suggested by a recent study, patterns of recovery might be different in hospitalized patients versus those in outpatient clinics [27]. Last, this study measured RNFLT, TRT, and ODA but did not separately analyze the ganglion cell layer (GCL) or inner plexiform layer (IPL). GCL thickness may serve as a potential indicator of permanent neuronal loss beyond RNFL changes alone [32]. Future studies should incorporate GCL segmentation to evaluate whether selective neuronal loss occurs independent of axonal changes.

The strength of our study was that all patients were enrolled when the IIH clinical diagnosis was first implied and before any therapy was initiated. Furthermore, its longitudinal design allowed for the monitoring of OCT optic parameters over the following year.

In conclusion, total retinal nerve fiber layer thickness measured by SD-OCT is a useful non-invasive adjunct tool for monitoring optic nerve recovery in newly diagnosed pediatric IIH. This study demonstrates that significant RNFL recovery occurs within 3 months in most children, with stabilization by 6–12 months to values approaching the normal range. Further studies including a large number of children with long-term follow-up are needed to stratify pediatric IIH based on SD-OCT parameters for sight-saving modification in diagnosis and treatment.

## Figures and Tables

**Table 1:** Patient demographics and clinical presentation.

	IIH Group N = 19	Control Group N = 25	*p*-Value
Age	12.9 ± 2.9	11.8 ± 3.4	0.4
N prepubertal/pubertal	9:10	10:15	
N (%) female	11 (57.8)	11 (44)	0.95
BMI	25.5 ± 9.7	22.3 ± 6.7	0.31
ICP cmH_2_O	37.3 ± 12.5		
Frisén score	2.9 ± 1.1		
Presentation (N: %)			
Headache	14 (73.6)	17 (68)	
TVO	7 (36.8)	0	
Tinnitus	4 (21.1)	0	
Nausea	8 (42.1)	8 (32)	
Vomiting	6 (31.5)	7 (28)	
Diplopia	4 (21.1)	0	
Photophobia	3 (15.7)	0	
Log MAR VA VA; (N)	0.09 ± 0.15 20/20 (N: 13) 20/32 (N: 4) 20/40 (N: 1) 20/80 (N: 1)	0.01 ± 0.04 20/20 (N: 22) 20/25 (N: 3)	0.029
VF:PMD (dB)	−5.14 ± 7.8	NA	

TVO: transient visual obscuration; PMD; perimetric mean deviation; BMI: body mass index; NA: not available; VA: visual acuity.

## Data Availability

The data that support the findings of this study are available on request from the corresponding author. The data are not publicly available due to privacy or ethical restrictions.
